# Custom 3D-printed split-type triflange implants for severe acetabular defects: mid-term clinical outcomes and biomechanical insights from finite element analysis

**DOI:** 10.3389/fbioe.2025.1702276

**Published:** 2025-11-26

**Authors:** Yu Guo, Dehong Feng, Xiaofeng Gu, Ling Wang, Yujian Ding, Yi Liu, Jialin Qin

**Affiliations:** Department of Orthopedics, The Affiliated Wuxi People’s Hospital of Nanjing Medical University, Wuxi People’s Hospital, Wuxi Medical Center, Nanjing Medical University, Wuxi, China

**Keywords:** 3D-printed, triflange cup, split-type, revision, acetabular defect, finite element analysis

## Abstract

**Background:**

Paprosky 3B acetabular defects challenge revision total hip arthroplasty (THA) due to conventional techniques’ high failure rates. This study evaluates midterm outcomes and biomechanical performance of 3D-printed split-type triflange acetabular cups for Paprosky 3B defects.

**Materials and methods:**

From 02/01/2017 to 10/30/2021, we retrospectively assessed 14 patients with Paprosky 3B defects using 3D-printed split-type triflange cups. Preoperative CT-based 3D models guided implant design with porous surfaces and optimized screw fixation. Clinical outcomes were assessed via Harris Hip Score (HHS) and Oxford Hip Score (OHS). Radiographic parameters and implant stability were analyzed. Biomechanical characteristics were evaluated through finite element analysis (FEA) under physiological loads representing single-leg stance, walking, and jogging (700N, 2800N, and 4200N).

**Results:**

At mean 74.2-month follow-up, HHS improved from 31.9 ± 8.5 to 82.9 ± 5.9 (p < 0.05) and OHS from 7.6 ± 2.3 to 35.4 ± 3.1 (p < 0.05). Anatomical hip center restoration was achieved with comparable postoperative and contralateral rotation center measurements. Radiographic analysis confirmed stable fixation in all cases without loosening. FEA revealed distinct biomechanical behavior between bone models. In both normal and osteoporotic models, stress concentrated at the superior flange screw fixation site and the superior acetabular rim. Under 700N loading, interfacial micromotion at all measurement points (P1-P3) remained below the 40 μm threshold for osseointegration. However, at higher loads (2800N and 4200N), P1 micromotion significantly exceeded this critical threshold in both models, reaching 122.861 μm and 131.244 μm respectively at maximum loading, while P2 and P3 maintained acceptable levels.

**Conclusion:**

Custom 3D-printed split-type triflange prostheses achieve excellent midterm functional restoration and biomechanical stability in Paprosky 3B defects. Key advantages include precise hip rotation center reconstruction, favorable stress distribution, and reduced intraoperative morbidity. Early partial weight-bearing is safe, though high-impact activities should await radiographic confirmation of osseointegration. Long-term validation of durability is warranted.

## 1 Introduction

Total hip arthroplasty (THA) remains the gold standard for end-stage hip diseases, significantly improving functional outcomes and quality of life. With the aging population and expanded indications in younger patients, global THA utilization continues to rise, exceeding 600,000 procedures annually in Europe, and is projected to surpass 1.4 million worldwide by 2030 (Manson and Schmidt, 2016; Ceddia et al., 2025). While modern implants demonstrate 10-year survival rates of 96% (Hunt et al., 2018), long-term revision rates escalate to 42% postoperatively at 25 years (Evans et al., 2019), primarily driven by aseptic loosening and periprosthetic joint infection (Kenney et al., 2019). These complications frequently culminate in progressive bone loss, posing formidable challenges for acetabular reconstruction.

The Paprosky classification system is widely used for categorizing acetabular defects, with type 3B (characterized by >60% bone loss, superior-medial hip center migration >3 cm, and frequent pelvic discontinuity) representing the most complex reconstruction scenario (Paprosky et al., 1994). Extensive bone loss may lead to pelvic discontinuity, complicating anatomical restoration and impeding stable implant fixation. To address this challenge, various revision techniques have been proposed, including oblong cups, jumbo hemispherical cups, bulk structural allografts, reinforcement rings/cages, trabecular metal augments, and cup-cage constructs (Di Laura et al., 2023; Sanghavi et al., 2024; Goriainov et al., 2021). However, comparative analyses of these techniques remain challenging due to inconsistent outcomes and notable complication rates, such as inadequate osseointegration, implant instability, mechanical failure, and gait abnormalities (Meding and Meding, 2023). Studies indicate that the 10-year re-revision rate following initial acetabular revision remains as high as 20%–36% (Deere et al., 2022; Abrahams et al., 2020), underscoring the limitations of current strategies in ensuring long-term stability for complex defects.

The advent of three-dimensional (3D) printing technology has ushered in a new era for managing complex acetabular defects. This technology enables the fabrication of patient-specific implants with controlled porous structures that mimic the elastic modulus of native bone, thereby mitigating the stress-shielding effect and promoting osseointegration. Emerging studies have demonstrated promising outcomes for 3D-printed acetabular components (Goodman and Engh, 2016). Our preliminary study (Ding et al., 2023) validated the clinical efficacy of 3D-printed integrated and split-type triflange acetabular cups for Paprosky III defects. Postoperative Harris Hip Score (HHS) improved from 28.6 to 83.8, with no mechanical failures observed over a 40.8-month follow-up period.

However, a critical concern with multi-component modular acetabular prosthetics is the risk of mechanical failure at the interfaces between parts (Strahl et al., 2023). Finite element analysis (FEA), a mature technology in computer-aided engineering, enables quantitative simulation of stress responses under physiological loads by constructing high-precision digital models, thereby providing theoretical foundations for optimizing prosthesis design. Recent biomechanical studies employing FEA have confirmed that patient-specific 3D-printed porous titanium augments for Paprosky type III defects yield superior stress distribution across the implant-bone construct compared to off-the-shelf augments, with significantly lower peak stresses in the augment, fixation screws, and surrounding bone under simulated gait and jogging loads (Ceddia et al., 2025). Nevertheless, the biomechanical behavior and underlying mechanisms of 3D-printed split-type triflange implants, particularly regarding stress concentration at the component interfaces and the risk of micromotion, remain unexplored.

In the present study, we retrospectively analyzed the early clinical outcomes of 14 patients with type 3B acetabular defects who underwent reconstruction using custom 3D-printed split-type triflange acetabular cups at our institution. Multi-load FEA was further performed on a representative case to biomechanically validate the performance of this novel implant design. To our knowledge, this is the first report to systematically combine clinical outcomes with computational biomechanics in investigating the efficacy of 3D-printed split-type triflange implants for acetabular reconstruction, specifically aiming to elucidate the mechanical factors and failure mechanisms at the implant interfaces.

## 2 Materials and methods

### 2.1 Patients selection

We retrospectively reviewed our institutional database to identify all consecutive patients who underwent acetabular reconstruction using 3D-printed split-type triflange implants for Paprosky type 3B defects (with or without pelvic discontinuity) between 02/01/2017 and 10/30/2021. The inclusion criteria were as follows: (1) failure of primary or revised THA; (2) acetabular bone loss classified as Paprosky type 3B. The exclusion criteria were: (i) active periprosthetic infection; (ii) tumor-related bone defects; (iii) follow-up duration less than 12 months. All study procedures adhered to the ethical principles of the Declaration of Helsinki and were approved by our hospital’s ethics committee.

### 2.2 Preoperative planning and prosthesis design

**FIGURE 1:**
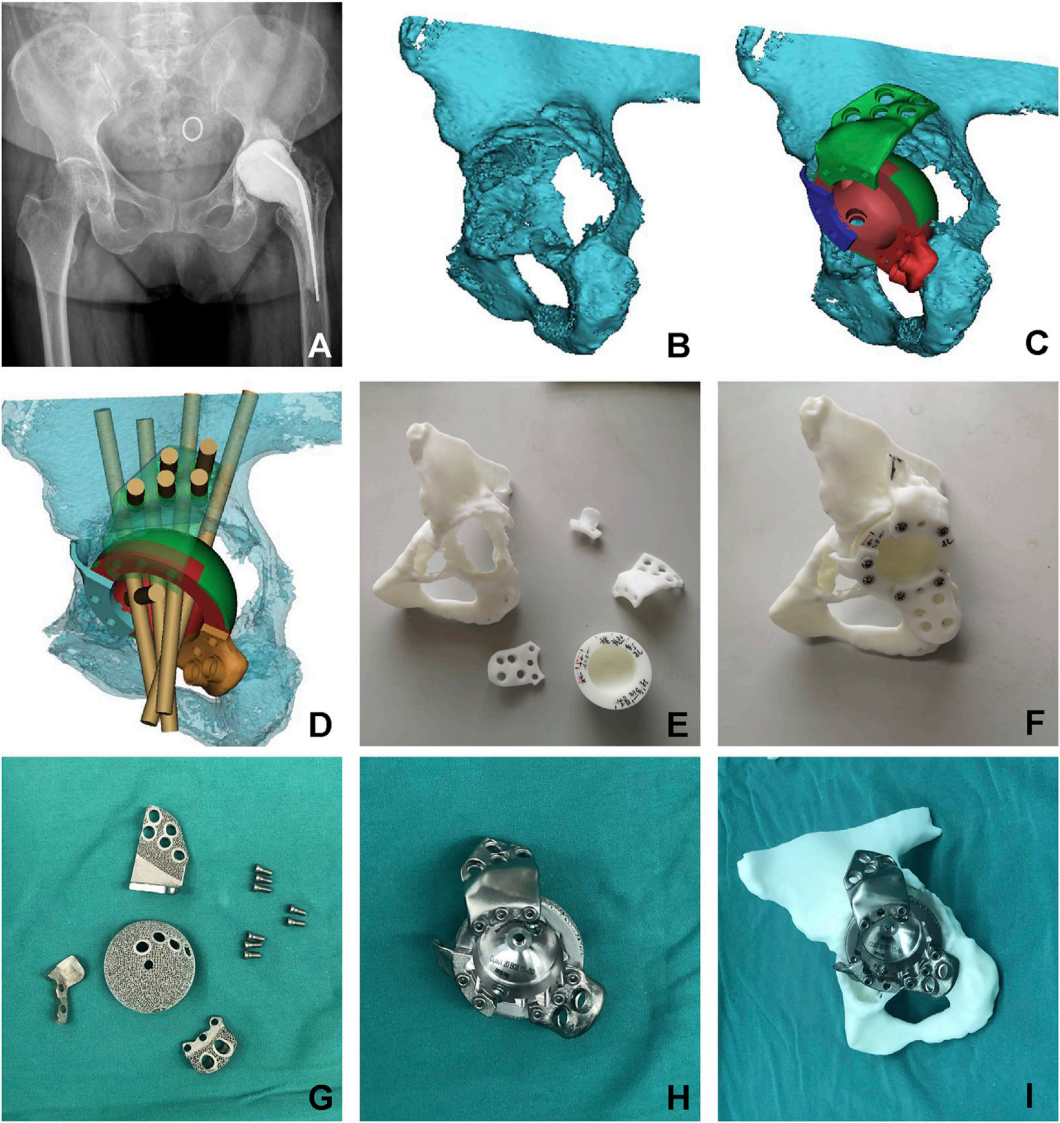
Preoperative planning for a 73-year-old female with Paprosky type 3B acetabular bone defect. **(A)** Preoperative radiograph demonstrating Paprosky type 3B acetabular bone defect with cement spacer *in situ*. **(B)** Three-dimensional reconstruction model of the acetabulum. **(C)** Computer simulation of prosthesis design. **(D)** Design of screw holes and screw trajectory. Screw hole configuration and trajectory planning. **(E)** 3D-Printed 1:1 scale resin model of the acetabular sefect and prosthesis prototype. **(F)** Preoperative simulation of prosthesis implantation using the resin model. **(G)** 3D-printed split-type triflange acetabular component with porous coating surface. **(H)** Assembled view of the acetabular cup component. **(I)** Final verification of prosthesis-acetabulum compatibility.

Pelvic CT scans (0.625 mm thickness) were saved in DICOM format and imported into Mimics software (Materialise, Belgium) for segmentation to generate a virtual 3D pelvic model. Based on this model, a digital implant design was created using computer-aided design (CAD) software (Magics, Materialise, Belgium) to match the patient-specific acetabular anatomy (Figures 1A–D). The prosthesis design principles included: (1) maximizing cup contact with residual anterior/posterior column bone, ensuring >60% porous surface contact between the cup-flange complex and host bone; (2) utilizing integrated metal augments to compensate for large central defects and expand bone-implant contact; (3) using the contralateral hip rotation center as the reconstruction reference; (4) optimizing screw configuration (number, position, length, and trajectory) to ensure stable implant fixation while mitigating the risk of neurovascular injury.

### 2.3 Preoperative validation with 3D-printed models

Following successful virtual surgical planning, physical validation was performed using life-size models fabricated from medical-grade polylactic acid with a Lite 450 HD printer (United 3D Tech, China). Models of the patient’s acetabulum and the split-type triflange prosthesis were accurately printed and assembled for preoperative simulation (Figures 1E,F). This process verified component alignment and implant seating while ensuring avoidance of neurovascular structures. When initial validation failed to meet established criteria, an iterative adjustment and re-simulation protocol was implemented until optimal fit was achieved.

### 2.4 Implant fabrication and post-processing

Upon confirmation of surgical feasibility, the final design was submitted to the manufacturer (Chunli Co., China) for implant fabrication using an Electron Beam Melting (EBM) system (Arcam Q10, Arcam AB, Sweden) (Figures 1G–I). The prosthesis was manufactured using Ti-6Al-4V powder with a particle size of 20–53 μm and a layer thickness of 50 μm, with the EBM process conducted at a scan speed of 1,300 mm/s. Post-processing included removal of excess powder particles using compressed air and ultrasonic cleaning, followed by stress-relief annealing at 800 °C for 2 h under an argon atmosphere with subsequent natural cooling. The final implant featured a 1.8 mm-thick porous surface with structural parameters of 600–650 μm pore size, 500–600 μm strut diameter, and 65%–70% porosity. The entire process required 2 days for design and 7 days for manufacturing, post-processing, and delivery.

### 2.5 Surgical procedure and postoperative recovery

**FIGURE 2:**
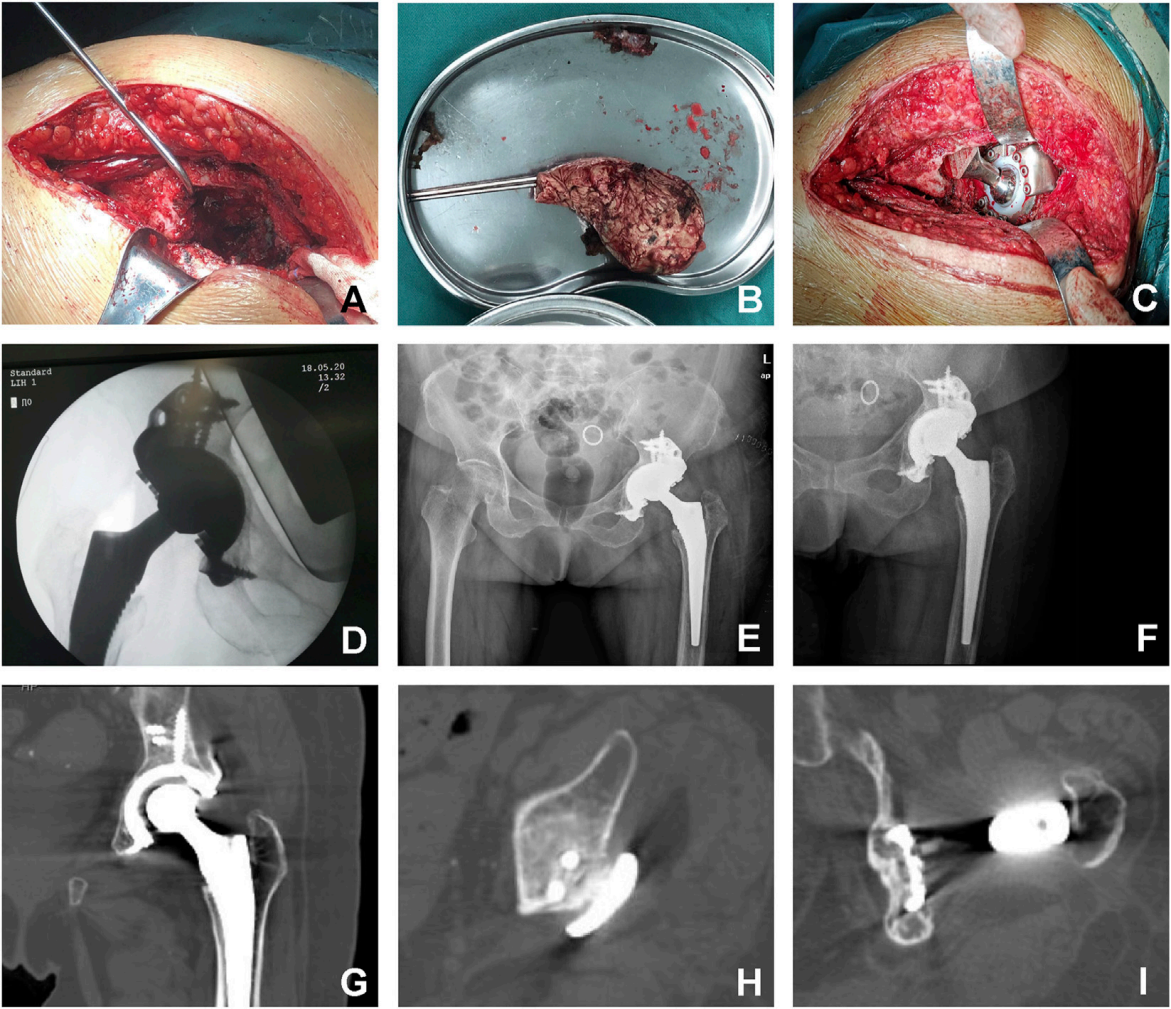
Surgical Procedure and Radiographic Follow-up. **(A)** Removal of cement spacer and exposure of acetabular bone defect morphology. **(B)** Extracted cement spacer. **(C)** Implantation of the 3D-printed triflange acetabular component. **(D)** Intraoperative fluoroscopy confirming optimal prosthesis positioning. **(E)** Postoperative day 3 anteroposterior radiograph. **(F)** 4-year postoperative anteroposterior radiograph. **(G–I)** Computed tomography (CT) scans at 4-year follow-up demonstrating osseointegration of the acetabular cup and flanges.

All procedures were performed by a senior orthopedic surgeon (D. F.) via a posterolateral approach under general anesthesia. After removal of the acetabular component, the bony acetabulum was exposed, and necrotic bone tissue was thoroughly debrided. Pulsed lavage (gentamicin 160,000 IU/500 mL saline) was applied, followed by impaction bone grafting with allogeneic bone granules in the defect area. The split-type triflange acetabular cup was implanted according to the preoperative plan, secured with locking screws through predefined trajectories, and the acetabular liner was fixed using a snap-fit mechanism (Figures 2A–D). Cementless or cemented femoral stems were selected based on femoral bone loss severity. Postoperative anticoagulation and antibiotic prophylaxis were administered routinely.

Physical therapy, including ankle pump exercises and lower limb isometric contractions, was initiated on postoperative day 1 to prevent venous thromboembolism. From 1 month postoperatively, patients began partial weight-bearing with a walker and progressed to hip stability and proprioception training. Full weight-bearing was permitted at 2 months.

### 2.6 Clinical and radiological evaluations

**FIGURE 3:**
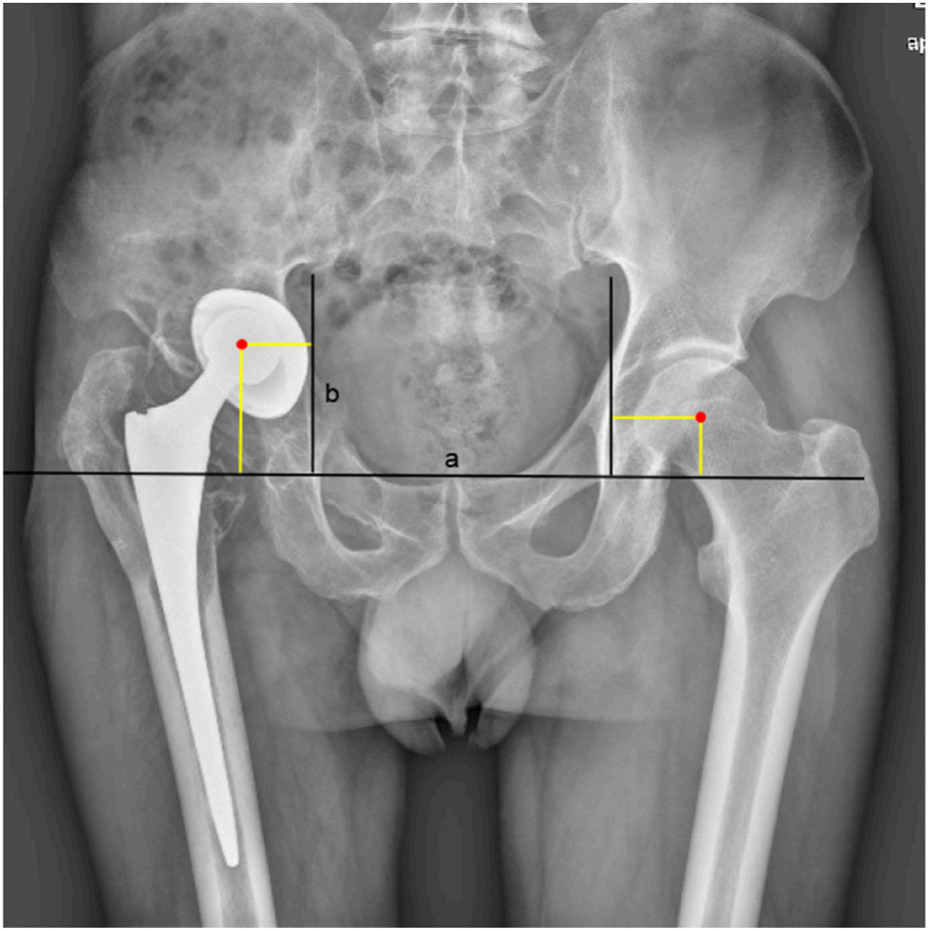
Radiographic Measurement of Hip Center of Rotation (COR). V-COR: Vertical distance between the femoral head center and the radiographic teardrop line (Line a). H-COR: Horizontal distance between the femoral head center and the vertical line through the inferior point of the teardrop (Line b).

Follow-up evaluations were conducted at 1, 3, 6, and 12 months postoperatively, then annually. At these visits, clinical outcomes were assessed using the HHS and OHS. All functional outcome assessments were performed by an independent research coordinator (J.Q.), who was not involved in the surgical procedures and was operationally independent of the surgical team. Radiographic assessments included anteroposterior pelvic radiographs or CT scans at each visit (Figures 2E–I). The hip center of rotation (COR) was evaluated preoperatively and postoperatively using the modified Ranawat method (Ranawat et al., 1980) on anteroposterior radiographs, measuring both the vertical (VCOR) and horizontal (HCOR) components (Figure 3). VCOR was defined as the vertical distance from the center of the femoral head to the inter-teardrop line, while HCOR represented the horizontal distance from the femoral head center to a vertical reference line passing through the inferior aspect of the teardrop. Osseointegration of acetabular components was evaluated using Moore’s criteria (Moore et al., 2006). Radiologic failure was defined by > 3 mm component migration, >5° inclination change, progressive radiolucent lines, or hardware fracture.

### 2.7 Acetabular cup position evaluation

**FIGURE 4:**
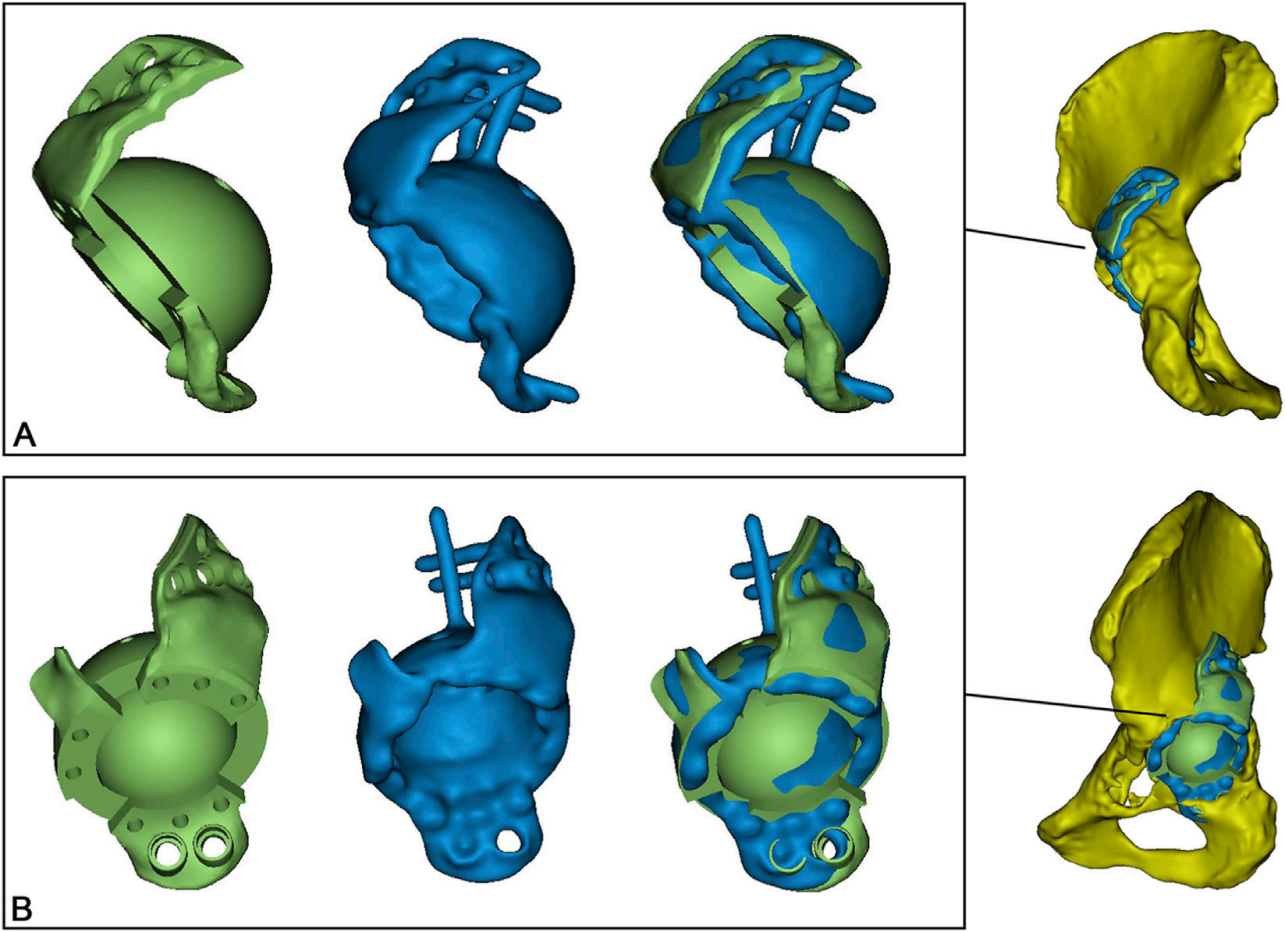
Acetabular Cup Position Evaluation. **(A)** Anteroposterior and **(B)** lateral views showing the preoperative planned (green) and postoperatively achieved (blue) implant positions.

Acetabular cup positioning was evaluated by blinded, non-surgical expert reviewers at our center. All patients received postoperative pelvic CT scans. The preoperative surgical plan model was registered and fused with the postoperative CT dataset using Geomagic software (Figure 4). This registration process was based on anatomical landmarks of the anterior pelvic plane (defined by the bilateral anterior superior iliac spines and the pubic tubercle) and the bilateral posterior superior iliac spines. The ischial spines served as auxiliary registration points when not obscured by metal artifact. A standardized anterior pelvic plane coordinate system was established within MIMICS software using the bilateral anterior superior iliac spines and the pubic tubercle. Specifically, the anterior pelvic plane was defined by the two anterior superior iliac spines and the midpoint of the pubic tubercles. The plane perpendicular to this was defined as the sagittal plane, and the longitudinal axis was defined by the line connecting the midpoint of the anterior superior iliac spines and the midpoint of the pubic tubercles. The COR was analyzed by decomposing its position into three orthogonal components: anteroposterior (AP), mediolateral (ML), and superoinferior (SI). Implant displacement was quantified by comparing the preoperative planned COR to the postoperative achieved COR, with a tolerance limit of 10 mm defined for clinically significant displacement. Furthermore, the cup inclination (INC) angle (defined as the angle between the acetabular axis and the sagittal plane) and anteversion (AV) angle (defined as the angle between the projection of the acetabular axis onto the sagittal plane and the longitudinal axis) were calculated according to the radiological definitions proposed by Murray (Murray, 1993). A tolerance limit of 5° was set for deviation in these cup orientation angles.

### 2.8 Finite element analysis

**FIGURE 5:**
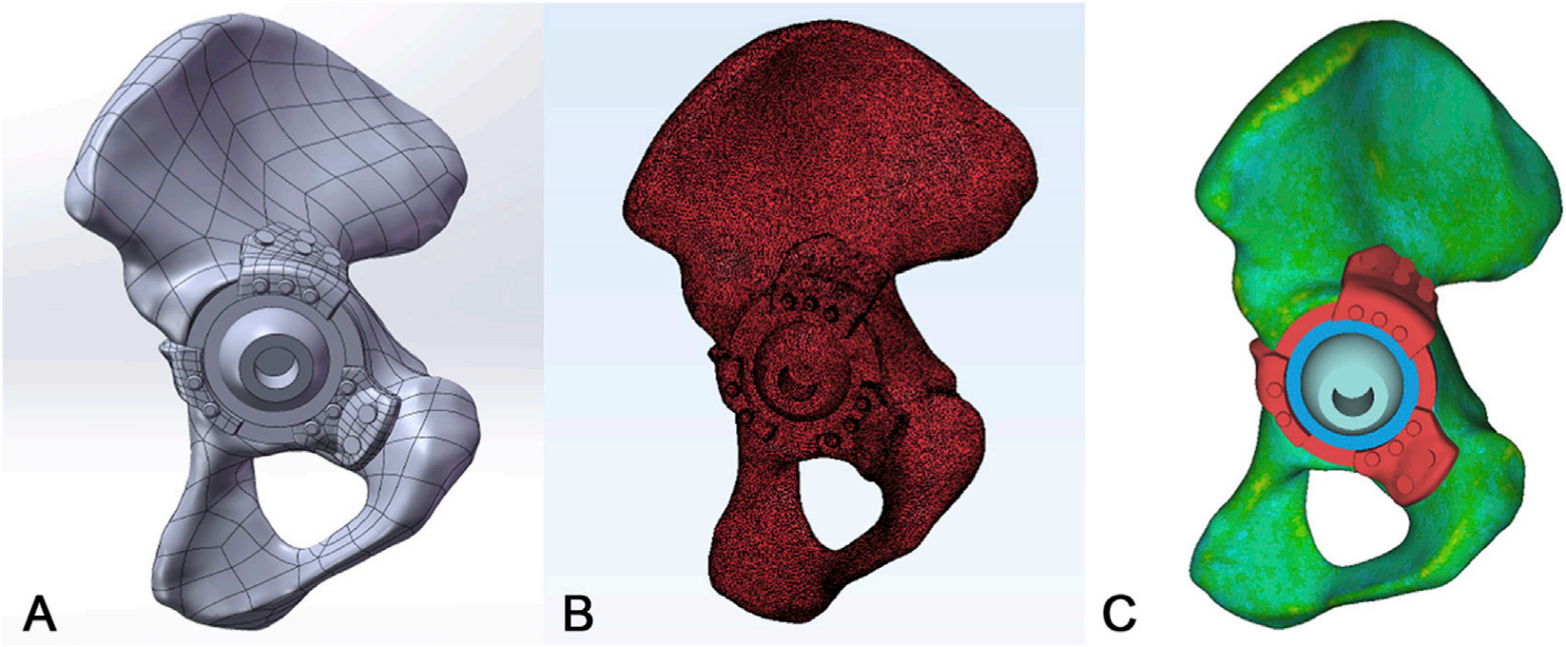
Finite Element Modeling and Material Property Assignment. **(A)** Finite element model of the acetabulum and implant. **(B)** Finite element mesh discretization of the acetabulum and implant. **(C)** Non-homogeneous, grayscale-dependent material properties assigned to bone.

A FEA was performed based on the anatomical geometry of a 54-year-old male patient (70 kg, 171 cm) who provided informed consent. A high-resolution CT dataset (slice thickness: 0.625 mm) was used to reconstruct a native pelvic 3D model. Surface optimization, including defect repair, smoothing, and precise surface fitting, was conducted using Geomagic Studio 2019 (Geomagic, Morrisville, NC, United States) while preserving the overall geometric fidelity. Then, the pelvic and implant models were then imported into SolidWorks 2024 (Dassault Systèmes, France), where Boolean operations were used to create a unified pelvic-implant assembly. The assembly was meshed in 3-matic 11.0 (Materialise, Leuven, Belgium)with a global element size of 1 mm. The quality of most surface elements, evaluated by the Height/Base ratio, satisfied the criterion of being greater than 0.3. A mesh sensitivity analysis demonstrated that reducing the element size below 1 mm resulted in variations of less than 5% in the FEA results (Zhu et al., 2025). Therefore, a uniform element size of 1 mm was adopted for the pelvic prosthesis model, generating a mesh comprising 658,460 elements and 373,711 nodes (Figures 5A,B). Material properties were then assigned to the pelvic bone. Non-homogeneous, grayscale-dependent material properties were assigned using Formulas 1, 2 within Mimics software (Figure 5C) (Iqbal et al., 2019; Moussa et al., 2020). The osteoporotic hip bone FEA was created by applying a 50% reduction to the elastic modulus of the normal bone.

$$\rho =6.9141e^{‐4}\times \text{HU}+1.026716$$ (1)

$$E=2017.3\times \rho^{2.46}$$ (2)

The final model was imported into ANSYS (Canonsburg, Pennsylvania, United States) for analysis, where the remaining material properties, as detailed in Table 1 (Liu et al., 2023; Li et al., 2022), were defined. The coefficient of friction was set to 0.5 for the bone-implant interface and 0.06 for the cup-femoral head interface. Bonded contact conditions were applied between all other components (Wang et al., 2022; Liu et al., 2023). To eliminate rigid body motion, fixed constraints were applied to the pubis and the superior ilium (Akrami et al., 2018).

**TABLE 1:** Mechanical properties of the materials used in the finite element models.

Components	Materials	Young’s modulus (MPa)	Poisson ratio
Pelvis	Inhomogeneous	-	0.300
3D printed flange	Titanium alloy	110,600	0.326
3D printed acetabular cup	Titanium alloy	110,600	0.326
Screws	Titanium alloy	110,600	0.326
Ceramic femoral head	Ceramics	350,000	0.220
Liner	Polyethylene	800	0.450

A vertical load was applied to the rotational center of the femoral head via a rigid plane, simulating the distal femur. Three loading conditions were modeled: 700 N (simulating single-leg stance), 2800 N (walking), and 4200 N (jogging), based on values from (Soloviev et al., 2023; Fu et al., 2018). Under these conditions, the stress distribution within the bone-implant system and the micromotion at the interfaces were analyzed.

### 2.9 Statistical analysis

Continuous data are primarily presented as mean ± standard deviation (Mean ± SD). To ensure a robust and complete descriptive account, the median and interquartile range (IQR) are also reported for critical outcome variables, offering complementary insights into the data distribution. Statistical analyses were performed using SPSS Statistics (Version 22.0). The normality of data distribution for all paired variables was assessed using both the Shapiro-Wilk test and visual inspection of Q-Q plots. Based on this assessment, paired comparisons were conducted using the paired t-test for normally distributed data or the Wilcoxon signed-rank test for non-normally distributed data. Statistical significance was defined as a two-sided p-value <0.05.

## 3 Results

### 3.1 Patient characteristics

A total of 14 patients (6 males, 8 females) met the inclusion criteria (Table 2). The mean follow-up duration was 74.2 months (range, 43–99 months). The mean age for revision surgery was 74.1 years (range, 61–87 years). Reasons for revision included aseptic loosening in 10 hips, metallosis-associated osteolysis in 2 hips, and two-stage reimplantation for periprosthetic infection in 2 hips. In this series, 12 out of 14 patients underwent first-time revision surgery, except for 2 patients who received a two-stage procedure due to periprosthetic infection. None of the patients died or was lost during follow-up.

**TABLE 2:** Demographics and outcomes of enrolled patients.

Variables	Value
Numbers of patients (hips)	14 (14)
Age (yr)	74.1 ± 8.1
Gender (male/female)	6/8
Side (right/left)	7/7
Body mass index (kg/m2)	23.1 ± 2.8
Diabetes (Yes/no)	5/9
Hypertension (Yes/no)	8/6
Osteoporosis (Yes/no)	6/8
Pelvic discontinuity (no. Of patients)	3
Clinical follow-up (mo)	74.2 ± 18.3
Reason for revision, n	
Aseptic loosening	10
Metallosis-associated osteolysis	2
Periprosthetic infection	2
HHS	
Preoperative	31.9 ± 8.5
Last follow-up	82.9 ± 5.9
p-value	<0.05
OHS	
Preoperative	7.6 ± 2.3
Last follow-up	35.4 ± 3.1
p-value	<0.05

HHS, Harris Hip Score; OHS, oxford hip score.

### 3.2 Clinical and radiographic outcomes

The HHS significantly improved from 31.9 ± 8.5 preoperatively to 82.9 ± 5.9 at final follow-up. The OHS also demonstrated marked improvement, increasing from 7.6 ± 2.3 to 35.4 ± 3.1 at the last follow-up. The VCOR of the operated side significantly improved from 50.1 ± 4.7 mm preoperatively to 17.5 ± 5.8 mm at the final follow-up. For the HCOR, the mean value on the operated side changed from 24.4 ± 6.7 mm preoperatively to 32.9 ± 4.6 mm at the final follow-up. Final follow-up measurements on the contralateral side were 16.8 ± 6.4 mm for V-COR and 33.6 ± 4.9 mm for HCOR, with no significant differences observed between the operated and contralateral sides for either parameter (Table 3).

**TABLE 3:** Radiographic outcomes of the hip COR.

Variables	Preoperative (mean ± SD/Median (IQR))	Postoperative (mean ± SD/Median (IQR))	p-value
VCOR (OS), mm	50.1 ± 4.7/49.7 (47.1–54.0)	17.5 ± 5.8/17.5 (14.4–19.7)	0.001
VCOR (CS), mm	16.8 ± 6.3/16.3 (11.7–20.1)	16.8 ± 6.4/16.4 (11.5–20.1)	0.664
p-value	0.001	0.146	
HCOR (OS), mm	24.4 ± 6.7/27.0 (20.0–28.5)	32.9 ± 4.6/31.2 (29.8–36.7)	0.001
HCOR (CS), mm	33.8 ± 5.2/32.1 (29.6–39.2)	33.6 ± 4.9/32.9 (29.7–37.1)	0.491
p-value	0.001	0.276	

OS, operated side; CS, contralateral side; COR, center of rotation; VCOR, the vertical distance between the COR, and the inter-teardrop line; HCOR, the horizontal distance between the COR, and the perpendicular line through the inferior point of the teardrop.

At the latest follow-up, anteroposterior pelvic radiographs demonstrated stable fixation without radiolucent lines around any of the 14 implants. The earliest evidence of bone ingrowth was observed on CT scans at the 12-month postoperative assessment. New bone formation was predominantly identified in stress-bearing regions, particularly the acetabular roof and areas adjacent to the superior flange and fixation screws. Notably, two cases exhibited osteolytic changes at the ischial and pubic flanges on CT imaging (Figure 2I), though follow-up examinations confirmed these lesions showed no signs of progression. No radiographic evidence of implant loosening, fracture, or migration was detected in any patient at final follow-up.

### 3.3 Complications

Two postoperative complications were documented during the study period. One patient developed persistent wound drainage, which resolved completely after surgical debridement. Another patient experienced dislocation 1 week postoperatively and was treated with closed reduction, followed by 4 weeks of percutaneous traction, with no recurrence. No cases of deep infection, thromboembolism, or implant failure were observed.

### 3.4 Accuracy of acetabular cup positioning

All implanted prosthetic components (100%) were positioned with a deviation of the COR within 10 mm from the planned position in all three anatomical planes (Table 4). The mean differences between the planned and achieved COR were 1.4 mm (95% CI: −0.6–3.6) in the AP plane, 0.1 mm (95% CI: −1.4–1.6) in the ML plane, and 0.3 mm (95% CI: −1.5–2.0) in the SI plane. For acetabular cup orientation, 9 out of 14 components (64.3%) had both INC and AV within 5° of the planned values. The mean planned INC was 40.1° (95% CI: 36.9–43.4), compared to an achieved mean of 41.1° (95% CI: 38.1–44.0), resulting in a mean difference of 1.0° (95% CI: −1.4–3.3). For anteversion, the mean planned angle was 14.9° (95% CI: 11.4–18.3), and the mean achieved angle was 16.4° (95% CI: 13.3–19.5), with a mean difference of 1.6° (95% CI: −1.5–4.6).

**TABLE 4:** Accuracy of acetabular cup positioning.

Case number	COR (AP shift) (mm)	COR (ML shift) (mm)	COR (SI shift) (mm)	ΔINC (°)	ΔAV (°)
Case 1	2.1	0	2.6	2.1	3.6
Case 2	2.2	5.3	0.7	3.6	**−11**
Case 3	0	2.2	−1.5	−1.5	4.8
Case 4	−3.1	−2.5	−2.4	3.9	4
Case 5	8.2	−4.3	−2.1	**9**	**−8.1**
Case 6	2.5	2.8	−3.5	−0.7	−1.6
Case 7	2.1	1.3	0	**−7**	**6**
Case 8	0	0	2.2	−0.2	−0.9
Case 9	−2.9	1.7	6.1	4.2	4.8
Case 10	1.5	−3.9	0.5	−0.7	2.7
Case 11	1.9	1.6	−3.2	3.5	3
Case 12	6.6	−2.1	−1.9	3.5	**5.5**
Case 13	−5.1	0	0.7	−3.3	**5.8**
Case 14	4.8	−0.7	5.6	−3	3.4

Discrepancies of more than 5° for cup angles between planned and postoperative positions are marked in bold. AP, anteroposterior; ML, mediolateral; SI, superoinferior; INC, inclination; AV, anteversion.

### 3.5 Finite element analysis results

**FIGURE 6:**
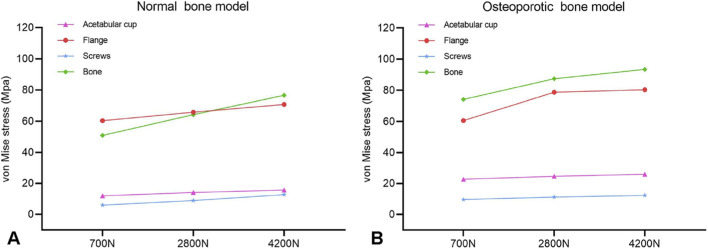
Variation of von Mises stress in various prosthetic components across loading conditions for the **(A)** normal bone model and **(B)** osteoporotic bone model.

This study evaluated the biomechanical performance of a custom acetabular implant in both normal and osteoporotic bone pelvises under varying load conditions (700N, 2800N, and 4200N) using finite element analysis. For both models, the maximum von Mises stress in all prosthetic components and the acetabular bone exhibited a linear positive correlation with the increasing load magnitude (Figure 6).

**FIGURE 7:**
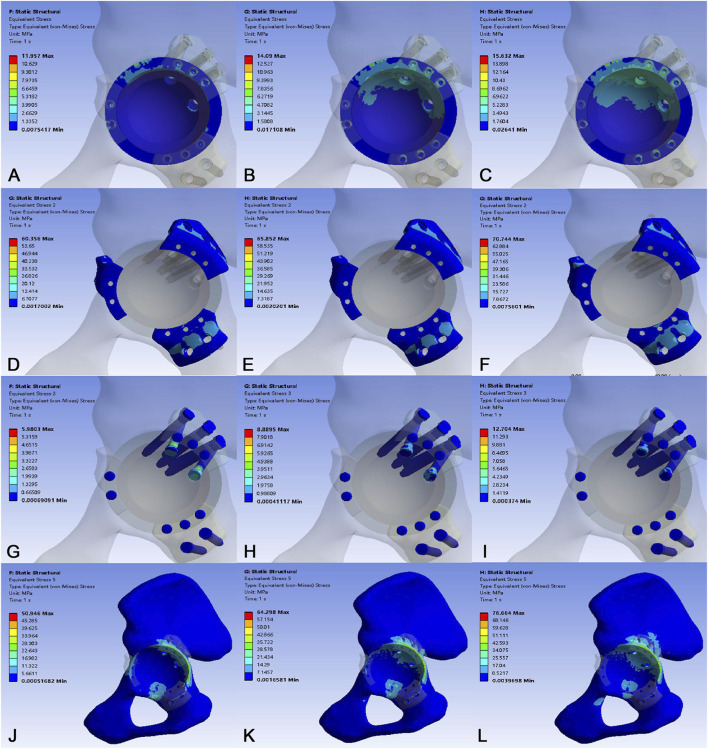
Stress distribution in the normal bone model under loads of 700 N, 2800 N, and 4200 N. **(A–C)** Stress distribution of the 3D-printed acetabular cup. **(D–F)** Stress distribution of the 3D-printed flanges. **(G–I)** Stress distribution of the fixation screws. **(J–L)** Stress distribution of the acetabular bone.

In the normal bone model (Figure 7), stress analysis revealed that the acetabular cup sustained peak stress at its superior region, rising from 11.957 MPa at 700N to 15.632 MPa at 4200N. The implant flange carried a higher load, with stress concentrating at the superior screw fixation site and varying from 60.356 MPa to 70.744 MPa. The fixation screws experienced maximum stress at the screw-flange interface, increasing from 5.980 MPa to 12.704 MPa. Crucially, the native pelvic bone exhibited the highest stress in the construct at the superior screw site, escalating from 50.946 MPa to 76.664 MPa. All recorded stress values remained safely below the yield strength of the titanium alloy, confirming a sufficient margin of safety under static loading conditions. Analysis of interface micromotion in the normal bone model revealed a load-dependent and location-specific response. Micromotion was most pronounced at location P1, increasing markedly from 22.985 μm at 700N to 122.861 μm at 4200N. In contrast, adjacent sites P2 and P3 exhibited significantly lower displacement, reaching only 6.194 μm and 16.587 μm, respectively, at 4200N. This resulted in P1 micromotion being 19.8 and 7.4 times greater than at P2 and P3 under the maximum load, indicating primary instability at the superior cup-bone interface (Table 5).

**TABLE 5:** Stress results and interface micromotion in a normal bone model under different loads.

Parameters	700N	2800N	4200N
Von mises stress (Mpa)			
Acetabular cup	11.957	14.090	15.632
Flange	60.356	65.852	70.744
Screws	5.980	8.889	12.704
Bone	50.946	64.298	76.664
Interface micromotion (µm)			
P1	22.985	82.866	122.861
P2	1.342	2.563	6.194
P3	4.897	10.560	16.587

**FIGURE 8:**
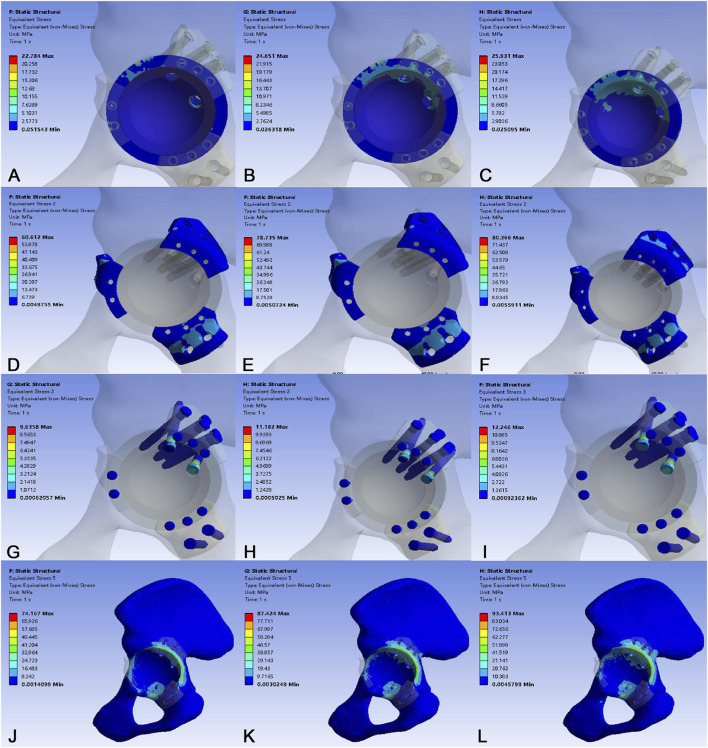
Stress distribution in the osteoporotic bone model under loads of 700 N, 2800 N, and 4200 N. **(A–C)** Stress distribution of the 3D-printed acetabular cup. **(D–F)** Stress distribution of the 3D-printed flanges. **(G–I)** Stress distribution of the fixation screws. **(J–L)** Stress distribution of the acetabular bone.

**FIGURE 9:**
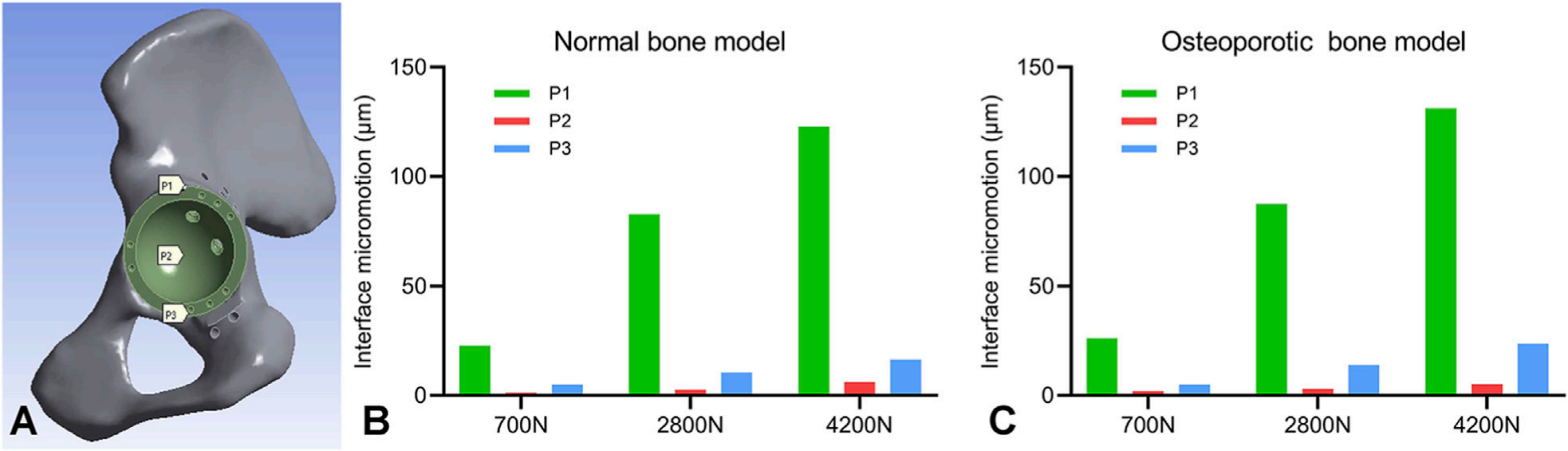
Acetabular cup interfacial micromotion under physiological loading. **(A)** Definition of the three measurement locations (P1, P2, P3) for micromotion quantification. Comparison of micromotion magnitudes at these locations under different loads for the **(B)** normal bone model and **(C)** osteoporotic bone model.

In the osteoporotic bone model (Figure 8), the overall stress pattern was similar, but the magnitudes were consistently higher. The acetabular cup sustained greater stress, peaking at 25.931 MPa. The flange and screw stresses were also elevated, with the flange reaching 80.366 MPa and the screws 12.246 MPa at 4200N. A critical finding was the significantly increased peak stress in the osteoporotic bone itself, which reached 93.413 MPa at 4200N compared to 76.664 MPa in the normal bone model (Table 6). Interface micromotion in this model demonstrated a similar load-dependent trend, with P1 reaching 131.244 μm at maximum loading. Notably, the micromotion at P3 (23.768 μm) was approximately 43% higher than in the normal bone model (16.587 μm), suggesting compromised interfacial stability in osteoporotic bone (Figure 9).

**TABLE 6:** Stress results and interface micromotion in an osteoporotic bone model under different loads.

Parameters	700N	2800N	4200N
Von mises stress (Mpa)			
Acetabular cup	22.784	24.651	25.931
Flange	60.612	78.735	80.366
Screws	9.635	11.182	12.246
Bone	74.167	87.424	93.413
Interface micromotion (µm)			
P1	26.346	87.470	131.244
P2	2.081	3.104	5.173
P3	4.875	14.033	23.768

## 4 Discussion

Revision arthroplasty for Paprosky type 3B acetabular defects remains highly challenging, particularly in cases with major segmental bone loss or pelvic discontinuity, where residual host bone fails to provide adequate support for conventional hemispherical cups (Di Laura et al., 2023). The integration of computer-assisted design and 3D-printed customized implants has emerged as a promising solution. Li et al. (2016) reported outcomes of 26 Paprosky 3B cases treated with 3D-printed custom cages, achieving a mean HHS of 82 at 67-month follow-up, though complications included 1 aseptic loosening, 2 infections, 1 dislocation, and 1 neurological injury. Goriainov et al. (2021) reported a 100% implant survival rate in 19 Paprosky 3B cases using 3D-printed triflange cups, with OHS improving from 8.6 to 35. Di Laura et al. (2023) documented a 5-year follow-up of 26 Paprosky 3B cases using 3D-printed triflange implants, showing a mean OHS increase from 8% to 32% and 92% osseointegration rates without loosening. In the present study, we reviewed the clinical outcomes of utilizing custom 3D-printed split-type triflange implants for reconstructing Paprosky 3B acetabular defects with a mean follow-up of 74.2 months. Our findings demonstrate that these implants offer a viable solution for severe acetabular bone loss, evidenced by a 100% survival rate, a statistically significant improvement in functional outcomes, and robust osseointegration without implant failure. Our intermediate-term results are encouraging and provide a rationale for the use of these implants.

Anatomic restoration of the hip COR is critical for biomechanical reconstruction. Medial or superior displacement of the COR compromises the mechanical efficiency of the gluteus medius, impairing abductor function (Fukushi et al., 2018). Superior displacement greater than 10 mm is specifically associated with elevated revision rates (Hendricks and Harris, 2006). Conversely, excessive lateral or inferior COR displacement increases gluteus medius tension, potentially leading to periarticular pain or neuropathic symptoms due to sciatic nerve traction. These risks underscore the importance of precise COR reconstruction. Traditional implants for Paprosky type 3B defects often result in COR mispositioning (Chang et al., 2021), whereas 3D-printed custom acetabular components demonstrate superior accuracy in restoring physiologic COR. Goriainov et al. (2021) reported mean horizontal and vertical COR deviations of 2 mm (range: 1–4 mm) and 2 mm (range: 0–3 mm), respectively, using 3D-printed monolithic triflange cups, compared to the contralateral hip. Fang et al. (2022) achieved postoperative VCOR and HCOR values of 20.8 ± 2.0 mm and 30.2 ± 1.6 mm, with <2 mm deviation from preoperative baselines. In this study, the COR was restored to within normal limits in all patients following revision arthroplasty. Postoperative VCOR and HCOR measurements demonstrated no significant differences from the contralateral side (discrepancies <1 mm). These outcomes confirm the technique’s efficacy in intraoperative reconstruction of the hip rotation center.

Existing literature on the positioning accuracy of custom implants is limited, often defining implant misalignment as a deviation of inclination/anteversion exceeding 10° or a COR deviation exceeding 5 mm (Baauw et al., 2015). This study systematically evaluated the positioning accuracy of custom acetabular implants by matching preoperative plans with postoperative CT scans. The results demonstrated encouraging outcomes in this case series: all prosthetic components (100%) were positioned within 10 mm of the planned COR across all three planes; and 64.2% of the components had both the cup INC and AV angles controlled within a 5° deviation from the planned values. Baauw et al. (2015) reported that among 16 revision total hip arthroplasties for Paprosky type 3 defects, 7 cases were malpositioned in one or more parameters: one in INC, three in AV, four in rotation, and five in COR. Durand-Hill et al. (2020) observed that 18 of 20 components (95%) had rotational deviations within 10°, and 11 components (58%) were positioned within 5° of the planned acetabular cup angles. Our results are comparable to these previous findings. Precise prosthetic positioning not only ensures anatomical reconstruction of the center of rotation and optimizes joint biomechanics but also creates favorable conditions for bone ingrowth, thereby establishing a solid foundation for achieving long-term clinical stability.

Paprosky type 3B defects are characterized by severe bone loss involving the acetabular dome and anterior/posterior columns, often accompanied by pelvic discontinuity, with residual host bone contact area typically <50% (often <30%) (Telleria and Gee, 2013). Surgical reconstruction of such defects should not only match physiological stress and transfer mechanical load but also restore the hip rotation center and hip joint function (Sanghavi et al., 2024). Traditional integrated multi-flanged cups represent a viable option, providing favorable initial biomechanical stability (Sershon et al., 2021). However, their intraoperative implantation often requires an extended incision and extensive soft tissue dissection, increasing risks of iatrogenic soft tissue injury or additional bone resection for proper seating. To address this limitation, (Roessler et al., 2019) developed a “modular augment-and-cage system” incorporating iliac/ischial flanges, obturator hooks, and bone defect augments to enhance intraoperative flexibility. This study proposes an optimized split-wing anatomically adapted cup design as a further development of this concept: the iliac, ischial, a nd pubic wings are individually fabricated based on patient-specific acetabular morphology and assembled intraoperatively with the acetabular cup. Pre-engineered screw trajectories accommodate anatomical variability, while avoiding neuro and vascular structures. This implant can be regarded as a customized “modular augment-and-cage system,” achieving minimized soft tissue disruption and bone resection without affecting the overall structural strength. However, Strahl et al. highlighted inter-component failure risks inherent to modular acetabular prostheses (Strahl et al., 2023). Consequently, we conducted finite element analysis to assess the biomechanical properties of the 3D-printed split-type triflange implants.

To our knowledge, this is the first such investigation of a 3D-printed split-type triflange acetabular prosthesis. The stress distribution pattern of the prosthesis in both normal and osteoporotic bone models corresponded with the directional vectors of hip joint forces, demonstrating progressive increases in von Mises stresses under escalating loads. In both models, stress concentrations localized at screw fixation points of the superior flange within the flange components. Elevated stresses emerged at the superior rim region of the acetabular cup, while maximum screw stresses occurred at the screw-flange interfaces. Under the maximum load (4200 N), the peak von Mises stress in the normal bone model measured 70.744 MPa in the flange, 15.632 MPa in the acetabular cup, and 12.704 MPa in the fixation screws. In contrast, the osteoporotic bone model exhibited a peak von Mises stress of 80.366 MPa in the flange, 25.931 MPa in the acetabular cup, and 12.246 MPa in the screws. The reported yield strength is 889–921 MPa for solid titanium alloy (Ti6Al4V) (Yang and Liu, 2016) and 263 MPa for porous titanium alloy (Noronha et al., 2024). Our data demonstrate that the peak von Mises stresses across all components in both models remained substantially below the yield strength threshold of porous titanium alloy. Our results confirm adequate structural safety margins under static loading conditions.

Stresses generated at the implant-bone interface and within their structural components may influence the biomechanical performance of the implant/bone system or potentially lead to failure during postoperative phases (Ma et al., 2013). The average yield strength of cortical bone near the acetabulum is 93.4 MPa (Fu et al., 2018). Our findings revealed that maximum bone stress occurred at the screw locations superior to the acetabular dome. Under simulated loading conditions of 700N, 2800N, and 4200N, the peak acetabular bone stress in the normal bone model remained well below the yield threshold of cortical bone. Notably, in the osteoporotic bone model, the corresponding peak bone stresses were substantially higher, reaching 74.167 MPa, 87.424 MPa, and 93.413 MPa, respectively. The peak stress recorded in the osteoporotic model under the maximum 4200 N load reached this critical yield threshold. These results suggest that normal periacetabular bone retains sufficient mechanical integrity to withstand early postoperative weight-bearing activities, including standing, walking, and jogging. However, in severely osteoporotic bone, the construct may transfer higher stresses to the periacetabular region, and postoperative weight-bearing should be postponed to prevent potential stress-induced fracture complications.

Previous studies have established a micromotion threshold of 40 μm for successful bone ingrowth in THA (Kaku et al., 2015). Our results demonstrated that under 700N loading, micromotion values at measurement points P1-P3 remained below this critical threshold in both bone models, confirming adequate interfacial stability during static weight-bearing. However, under walking and jogging conditions (2800N and 4200N), while P2 and P3 maintained subthreshold micromotion, point P1 in both models significantly exceeded the critical threshold, indicating potential instability at the superior fixation site. Numerical results indicated that intraoperative attention to superior dome fixation is particularly crucial for achieving initial stability, consistent with previous studies (Ghanem et al., 2020; Von Hertzberg-Boelch et al., 2021). Based on these findings, we recommend early partial weight-bearing rehabilitation for 3D-printed split-type triflange prostheses, while deferring high-impact activities such as jogging until radiographic confirmation of osseointegration, especially in patients with compromised bone quality. Our clinical outcomes validate this approach. All 14 patients achieved full weight-bearing by 2 months postoperatively, with no cases of implant loosening, migration, or fracture at final follow-up. Although computed tomography revealed limited osteolytic changes in two cases, these remained non-progressive and did not affect overall implant stability. The favorable clinical results may be attributed to several implant design features. The 65%–70% porosity three-dimensional trabecular structure provides an optimal environment for bone ingrowth (Migaud et al., 2019), while the one-piece molding process eliminates risks of coating delamination observed in traditional cementless implants. However, the specific performance of this porous structure in elderly patients with potentially diminished bone healing capacity requires further investigation through larger, long-term studies.

This study has several limitations that must be acknowledged. The retrospective design and small sample size limit the generalizability of the findings. Furthermore, the absence of direct comparisons with conventional monolithic triflange prostheses prevents a comprehensive assessment of this innovative implant’s relative performance. Although finite element analysis provides valuable biomechanical insights, the lack of laboratory mechanical testing and cadaveric validation may affect the translational accuracy of the computational results when compared to direct experimental evidence. Ultimately, the stress concentration at modular junctions requires longer-term data to validate its durability under real-world repetitive loading, despite the safety margin indicated by our mid-term and idealized analyses.

## 5 Conclusion

This study evaluated clinical and radiographic outcomes following reconstruction of Paprosky type 3B acetabular defects using customized 3D-printed split-type triflange acetabular implants in a patient population with a mean BMI within normal range. After 3–8 years of follow-up, favorable midterm outcomes were demonstrated including high implant survivorship, significant clinical improvement, and low complication rates, with biomechanical safety further validated by FEA. We recommend initiating early partial weight-bearing rehabilitation while deferring high-impact activities until radiographic confirmation of osseointegration. Based on these findings, we believe that this implant is a reliable and effective solution for severe acetabular defects.

## Data Availability

The original contributions presented in the study are included in the article/supplementary material, further inquiries can be directed to the corresponding author.
